# On the Move-Sensitive Fluorescent Aptassay on Board Catalytic Micromotors for the Determination of Interleukin-6 in Ultra-Low Serum Volumes for Neonatal Sepsis Diagnostics

**DOI:** 10.1021/acssensors.2c01635

**Published:** 2022-10-05

**Authors:** José Gordón, Luis Arruza, María Dolores Ibáñez, María Moreno-Guzmán, Miguel Ángel López, Alberto Escarpa

**Affiliations:** † Department of Analytical Chemistry, Physical Chemistry and Chemical Engineering, University of Alcalá, Ctra. Madrid-Barcelona, Km. 33.600, Alcalá de Henares, 28802Madrid, Spain; ‡ Department of Neonatology, Instituto del Niño y del Adolescente, Hospital Clínico San Carlos-IdISSC, 28040Madrid, Spain; § Clinical Laboratory Department, Instituto de Investigación Sanitaria San Carlos (IdISSC), 28040Madrid, Spain; ∥ Department of Chemistry in Pharmaceutical Sciences, Faculty of Pharmacy, Complutense University of Madrid, Plaza Ramón y Cajal s/n, 28040Madrid, Spain; ⊥ Chemical Research Institute “Andres M. Del Rio”, University of Alcalá, 28871Madrid, Spain

**Keywords:** micromotors, on-the-move aptassay, graphene, fluorescence microscopy, interleukin-6, neonate sepsis

## Abstract

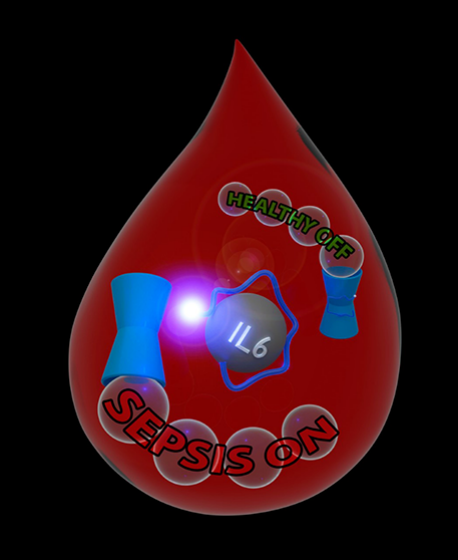

A graphene oxide/nickel/platinum nanoparticle micromotor (MM)-based fluorescent aptassay is proposed to determine interleukin-6 (IL-6) in serum samples from low-birth-weight infants (gestational age of less than 32 weeks and birthweight below 1000 g) with sepsis suspicion. In this kind of patients, IL-6 has demonstrated good sensitivity and specificity for the diagnosis of sepsis, both for early and late onset sepsis. The approach was based on the adsorption of the aptamer for IL-6 tagged with 6-FAM as a fluorescent label (Apt_IL-6_, λ_em_ = 520 nm) on the graphene oxide external layer (MM_GO_–Apt_IL-6_) inducing fluorescence quenching (OFF state) and a subsequent on-the-move affinity recognition of IL-6 from Apt_IL-6_ (IL-6–Apt_IL-6_ complex) recovering the fluorescence (ON state). An aptamer against IL-6 was selected and developed by the systematic evolution of ligands by exponential enrichment technology. This approach displayed a suitable linear range of 0.07–1000 pg mL^–1^ (*r* = 0.995) covering the cut-off and clinical practice levels, allowing direct determination without any dilution and simplifying the analysis as well as exhibiting an excellent sensitivity (LOD = 0.02 pg mL^–1^) in ultralow volumes of diagnostic clinical samples (2 μL). A high agreement between IL-6 levels obtained from our MM-based approach and the method used by the Hospital was obtained (relative error < 3%). The MM-based aptassay is competitive in comparison with that of the Hospital, in terms of a significant reduction of the sample volume (15 times less) and enhanced sensitivity, employing similar analysis times. These results position MM technology with enough potential to achieve high sensitivities in low sample volumes, opening new avenues in diagnosis based on low sample volumes.

Micromotor (MM) technology is in the front of biosensing research.^1−4^ It is a microdevice that transforms the energy obtained from a chemical reaction or an external stimulus into kinetic energy through its autonomous movement along a solution.^5^

Considerable efforts have been devoted to developing a chemically powered MM based on surface catalytic decomposition of a fuel solution, usually hydrogen peroxide. The continuous movement of a synthetic MM through the samples and the modulation of mass transport resulting from the locomotion of bubble-propelled MM significantly enhance the interactions of the MM sensing surfaces with the target analytes,^6^ thereby improving the recognition efficiency and offering on-the-move recognition of specific biomolecular interactions.^7^

From an analytical perspective, motion-based sensing approaches possess several advantages such as the reduction of the overall analysis time (through efficient mixing) and efficient collective operation in ultralow sample volumes (through the reduction of the size of the microsensor). Besides, the autonomous movement leads to a dynamic collective search for the analyte, improving the sensitivity of the assay as well as the kinetics. Tubular MMs are commonly prepared by template-based electrodeposition of polymers,^8^ metals,^9^ and carbon nanomaterials,^10^ and they present a surface that can be used for further functionalization with enzymes,^11^ antibodies,^12,13^ or aptamers.^14^ Particular attention has been given to tubular catalytic MM that can reach ultrafast speeds for on-the-move immunoassays.^7^ In addition, one of the inherent characteristics of MMs with high analytical potential is the exploration of their collective behavior for biosensing purposes, especially in low sample volumes. We think that this dimension is still unexplored.^15^

Because of the MM features discussed above, one of the most pertinent analytical applications of MM is their use in biosensing approaches for clinical diagnostics when the sample is hardly available. One example of high significance is neonatal sepsis diagnostics, where sample availability is scarce, especially in those babies with very low birth weight due to their low blood volume.

Sepsis is a systemic response to an infection caused by pathogens, viruses, bacteria, and parasites.^16^ Along with other diseases, it is one of the main causes of death in the world and one of the main problems that need to be solved in hospitals, especially in the intensive care unit (ICU), where it is necessary to begin treatment as soon as possible to decrease the chances of death.^17^ Currently, the gold standard for diagnostic is the blood culture, which is based on the presence of pathogens in the bloodstream.^18^ This technique needs relatively large volumes of sample and long analysis times besides displaying low sensitivities in neonates due to the irregularity of bacteremia and low sample volume, causing failure in the detection of sepsis.^19−21^ This entails the initiation of antibiotic therapy before confirmation of the diagnosis, with its potential side effects being the possibility of development of antibiotics resistance and the increment of healthcare costs.^22,23^ This challenge becomes even more complicated, if possible, in the diagnosis of neonatal sepsis, especially in very low-birth-weight infants who have a very small blood volume and given their increased vulnerability to infection due to the immaturity of their immune system. That is why ICU care for these patients is more expensive to save the incipient life.^24^

The diagnosis of sepsis is extremely complicated today and still far from being addressed at the exact moment of sepsis development and with the required reliability. Blood biomarkers constitute an additional aid in the diagnosis due to their increase in the early phases of the infection when signs and symptoms are not so evident. However, despite efforts, to date, there is no single ideal biomarker that can be considered as one with rapid results with perfect sensitivity and specificity along with low blood volume requirements for specific populations such as preterm neonates. Thus, the use of a panel of biomarkers has been proposed to increase diagnostic reliability. To obtain this great milestone of multiplexed analysis, it is necessary to look first at new technologies and develop alternative approaches to improve the determination of individual biomarkers.

Within the large number of biomarkers for sepsis diagnosis, around 250,^25^ interleukin-6 (IL-6) is one with the best sensitivity and specificity. Protein IL-6 is a cytokine synthesized from fibroblasts, endothelial cells, T-lymphocytes, and monocytes^26,27^ composed by 184 amino acids and a molecular weight of 21–26 kDa.^28^ IL-6 serves as a mediator in the inflammation process, playing an important role in some diseases^29−31^ and being involved in physiological and immunological processes. The reference value of IL-6 in the organism is 10 pg mL^–1^, and levels above 20 pg mL^–1^ are related to a positive diagnosis.^32^ One of the main advantages of IL-6 is that it increases its concentration after 2–4 h of exposure to infection, so it can be considered as a very early sepsis biomarker.^33^ Hence, the sensitive and quick determination of IL-6 allows more timely decisions. Metanalysis shows that IL-6 has a diagnostic performance similar to that of procalcitonin and superior to that of the C-reactive protein.^26^ Moreover, it can differentiate between sepsis and non-infectious inflammatory response. In neonates, IL-6 has demonstrated good sensitivity and specificity for the diagnosis of sepsis after prolonged rupture of membranes^34^ with improved performance when combined with other biomarkers, both for early and late onset sepsis.^35,36^

IL-6 has been determined by different approaches, such as chemiluminescent immunoassay,^37,38^ colorimetric immunoassay,^39^ electrochemical immunosensor,^40−45^ liquid-gated field-effect transistor,^46^ fluorescence immunoassay,^47−53^ and surface plasmon resonance biosensor,^54−56^ and with the use of aptamers (micro-array, impedance, and UV–vis spectrophotometry)^37,38,57−60^ among others.

While MMs functionalized with antibodies have previously been used in the diagnosis of neonatal sepsis for C-reactive protein^61^ and procalcitonin (PCT),^12^ it has been considered pertinent also to explore the possibilities of aptamers on board on MM technology for this type of diagnosis due to their high stability and the great versatility they present to carry out multiplexed assays, all without losing their selectivity and sensitivity.

Aptamers are single-stranded nucleic acids that are capable of specifically recognizing their target molecules with high affinity.^62^ Aptamers are generally obtained from oligonucleotide libraries using an in vitro method called systematic evolution of ligands by exponential enrichment (SELEX)^63^ that allows selecting that oligonucleotide from the library that binds with more affinity to the target molecule. These oligonucleotides have a central region of between 25 and 90 nucleotides and a random sequence and two flanking regions of known sequence that allow PCR amplification of the selected oligonucleotides. In many respects, the enriched population of aptamers could resemble polyclonal antibodies, and the individual aptamers derived from the population could resemble monoclonal antibodies. Aptamers, based on their three-dimensional structures, can bind to a wide variety of targets, such as antibiotics, hormones, peptides, proteins, or even entire cells or complex multimeric structures.

From the diagnosis point of view, aptamer-based biosensors, or so called aptasensors,^54^ make use of aptamers as biorecognition elements. Compared to antibodies, aptamers can be produced by chemical synthesis and are therefore less expensive to manufacture, have less variability between batches, and have very controlled post-production modification with no loss of activity. Also, aptamers are very resistant to degradation or denaturation, are easily labeled without loss of function, are smaller in size, and present the possibility of easily performing homogeneous assays, enabling quick and simple testing platforms. Over the last decade, aptamers have been used in numerous diagnostic platforms for the detection of various analytes ranging from small molecules to much more complex targets.^64−66^

In this work, we propose for the first time an aptassay approach based on the use of catalytic MMs for the determination of IL-6. To this end, an OFF–ON biosensing approach based on aptamers specifically designed for IL-6, using SELEX technology, has been developed. The detection principle has been designed through an on-the-move approach using an MM constructed with an outer GO layer due to its excellent quenching properties and with a PtNP inner catalytic layer for efficient propulsion. The analytical applicability of these pioneer MMs for IL-6 biosensing have been demonstrated by analyzing samples from very low-birth-weight neonatal patients with suspected sepsis, previously diagnosed.

## Experimental Section

### Reagents

The sequence of the IL-6 aptamer was reconstituted in phosphate-buffered MgCl_2_ (PBMgCl_2_) (0.1 M Na_2_HPO_4_ (99%), 0.1 M NaH_2_PO_4_, and 1 mM MgCl_2_ from Sigma-Aldrich (Madrid, Spain) pH 7.5. The protein IL-6 was purchased from Abcam (Cambridge, UK) and diluted in phosphate-buffered saline (PBS) buffer solution pH 7.5 (0.1 M Na_2_HPO_4_ (99%), 2.7 mM KCl (99%) from Scharlau (Madrid, Spain); 0.1 M NaH_2_PO_4_, 0.138 M NaCl (99%) from Panreac (Madrid, Spain).

Graphene oxide (GO) (4 mg mL^–1^ dispersion in H_2_O), hydrogen peroxide (30% v/v), H_2_SO_4_, Na_2_SO_4_, H_2_PtCl_6_, nickel(II) sulfamate tetrahydrate (H_4_N_2_NiO_6_S_2_), nickel(II) chloride hexahydrate (Cl_2_Ni–6H_2_O), isopropanol, and ethanol were purchased from Sigma-Aldrich. Boric acid (99.5%) was purchased from Fluka. Bovine serum albumin (BSA) was purchased from Sigma-Aldrich and dissolved in PBS. Polycarbonate (PC) membranes were purchased from Whatman (Maidstone, UK). All chemicals used were analytical-grade reagents, and deionized water was obtained from a Millipore Milli-Q purification system (18.2 MΩ cm at 25 °C).

### Aptamer Design and Synthesis

The sequence of the IL-6 aptamer was synthesized by Aptus Biotech with Alexa488. The initial aptamer library was chemically synthesized and HPLC-purified by IBA Lifesciences (Goettingen, Germany). The library consisted of a pool of 76 nt-long oligonucleotides with a central, 40 nt-long random region and two-flanking primer-binding regions of 18 nt-long each with a fixed sequence. For the selection of aptamers, recombinant human IL-6 (Abyntek Biopharma) fused to histidine at the N-terminal end was immobilized on the Ni-NTA Agarose resin (QIAGEN). The enrichment of the populations obtained after the three selection rounds for IL-6 were analyzed by real-time PCR. Next, massive sequencing (NGS - Illumina) was performed, and the sequences of the populations enriched in specific aptamers against IL-6 were identified by Aptasuite.^67^ The aptamer against IL-6 (IL-6R1) was finally synthetized with 6-FAM as the fluorescent label at 5′ with the aim of performing the aptassay procedure.

### Samples

The synthetic human serum from human male blood type AB was obtained from Sigma-Aldrich (Madrid, Spain). Blood samples were obtained from neonates admitted to the neonatal ICU (NICU) at Hospital Clínico San Carlos (Madrid, Spain). Inclusion criteria were suspected late-onset sepsis, gestational age of less than 32 weeks, and birthweight below 1000 g. A control group of babies with similar gestational age and birthweight, but without sepsis, was included for comparison. Late-onset sepsis was suspected by the presence of compatible symptoms (apnea, lethargy, thermal instability, hemodynamic and respiratory deterioration, etc.) and analytical signs of infection (e.g., altered white cell count, elevated acute phase reactants such as CRP, PCT, or IL-6) after 72 h of life and confirmed by a positive blood culture. The research protocol was approved by the local Ethics Committee, and parental informed consent was obtained in all cases before the inclusion in the study. Samples for investigation were collected only if a blood extraction was to be done as part of routine NICU care and not for the sole purpose of the present study. Samples were obtained by venipuncture.

### Apparatus

An electrochemical station μ-Autolab Type III (Eco Chemie, Utrecht, Holland) was used for template-assisted electrochemical deposition of the MM. An inverted optical microscope (Nikon Eclipse 80i upright micro-scope), coupled with an objective (Nikon S Fluor 20X/0.75 DIC M/N2, ∞/0.17, WD 1.0), a DAPI 5060C fluorescence filter (λ_ex_, 377 nm; λ_em_, 447 nm), a Hamamatsu digital camera C11440, and NIS Elements AR 3.2 software, was used for capturing images and movies. Advanced VortexMixer-ZX3 from VWR and Thermosaker TS-100C from Biosan were used for incubation stages. Magnetic block DynaMag-2 obtained from ThermoFisher was used for the handling of magnetic MMs. Scanning electron microscopy (SEM) images were obtained with a JEOL JSM 6335F instrument, and X-ray analysis was performed using an energy-dispersive X-ray spectroscopy (EDX) detector attached to an SEM instrument.

### Electrosynthesis of Graphene–Nickel–Platinum Nanoparticle MMs

**Figure 1:**
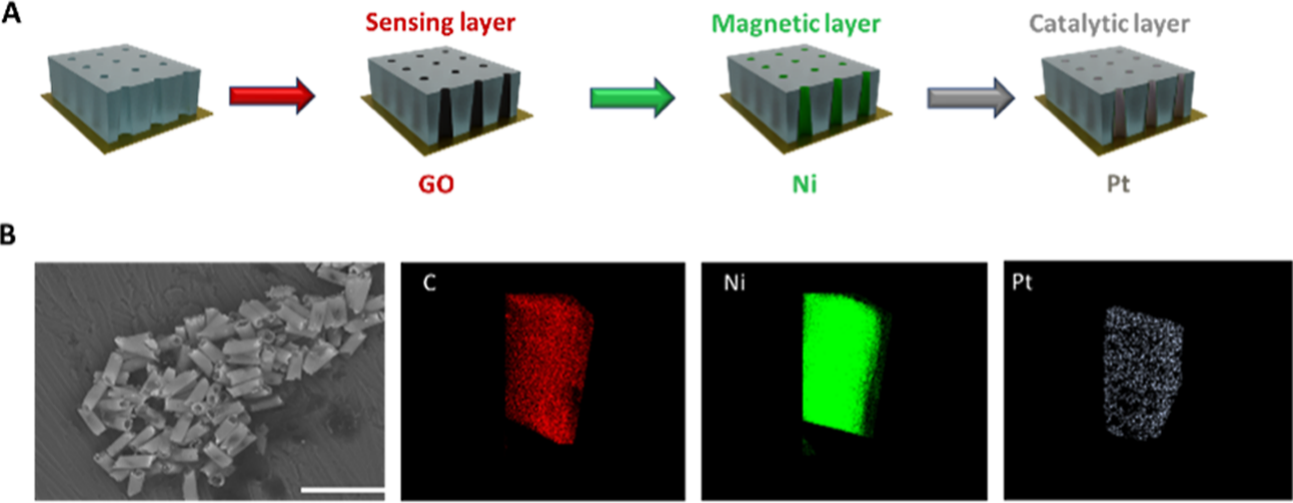
Schematics of the electrosynthesis of each MM layer: GO; Ni; and PtNPs (A). SEM images and EDX analysis (B) of the MM. Scale bar (B): 30 μm.

MMs follow a protocol based on electrodeposition above a PC membrane (see Figure 1A). In a previous step, the S4-branched side of 5 μm-diameter conical pores of a PC membrane was treated with a sputtered thin gold film to perform as a working electrode. The system was based in a Teflon cell with aluminum as an electrical contact to the working electrode and the membrane assembled in the center of the system. This synthesis was based on the electrodeposition of GO (GO layer), nickel (Ni layer), and platinum nanoparticles (PtNP layer) as successive layers. First, the outer layer based in carbon compounds was synthesized by the reduction of a solution of GO, 0.5 mg mL^–1^, 0.1 M H_2_SO_4_, and 0.5 M Na_2_SO_4_ previously dispersed in an ultrasonic bath for 15 min, using cyclic voltammetry through 10 cycles (+0.3 to −1.5 vs Ag/AgCl (3 M KCl), at 50 mV S^–1^). Second, the intermediate layer based on Ni was plated inside the reduced carbon layer through two different processes. First, 10 pulses of −20 mA were applied for 0.1 s to generate nucleation spots, followed by a constant current of −6 mA for 300 s to grow the nickel layer. Third, the inner layer was based on Pt deposited by amperometry at −0.4 V for 750 s by an aqueous solution containing 4 mM H_2_PtCl_6_ in 0.5 M boric acid. Once the depositions of the three materials were finalized, the sputtered gold layer membrane was gently hand polished with 0.3–1 μm alumina slurry. Successive washing of the MM was performed using a magnet-holding block with CH_2_Cl_2_ (3 washes) for 15 min to completely release the MM, and with isopropanol (10 min, 3 times), ethanol (5 min, 2 times), and water (5 min, 1 time) to get a neutral medium. All MMs were stored in ultrapure water at 4 °C when not in use. The template preparation method resulted in reproducible thousands of MMs with similar size and shape using a single membrane.

### IL-6–MM Aptassay

Preparation of an aptamer-modified MM (MM_GO_–Apt_IL-6_) as a previous reagent to perform the on-the-move aptassay was accomplished by deposition of 25 μL of a 0.4 μM specific IL-6 aptamer solution into a test tube together with 25 μL of MM suspension followed by a mechanical stirring incubation for 45 min at 25 °C in PBMgCl_2_ and three washes with PBS to remove all the material not reacted.

In a second step, MM_GO_–Apt_IL-6_ was used for on-the-move recognition of IL-6 molecules in a cocktail solution (2 μL of total volume) that contained the IL-6 sample without dilution or the standard dissolved in PBS with BSA (5%) and H_2_O_2_ (2%) for 30 min. Subsequently, the solution was deposited in an ELISA microwell and measured with a fluorescence microscope with a B2-A fluorescence filter (λ_ex_, 470 nm; λ_em_, 520 nm). The signals obtained were analyzed by the software predefined by the microscope and fitted through the equation
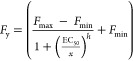
*F*_max_ and *F*_min_ are the maximum and minimum fluorescence intensity values of the calibration graph, respectively, *h* is the hill slope, and EC_50_ is the value of the analyte concentration corresponding to 50% of *F*_max_. The limit of detection (LOD) and limit of quantification (LOQ) were also calculated through the 3 S/N and 10 S/N criteria, where S is the standard deviation (*n* = 10) of the fluorescence using the lowest protein concentration used in the calibration and N is the slope of the linear regression obtained from the linear calibration plot.

## Results and Discussion

### MM-Based Aptassay Approach

Figure 1 shows a scheme for MM electrosynthesis (A) and characterization of GO/Ni/PtNP MMs using SEM images and EDX (B). Tubular magneto-catalytic MMs were synthetized by electrodeposition of three specific functional layers: (i) outer GO layer for free aptamer fluorescence quenching, (ii) intermediate Ni magnetic layer for magnetic guidance, and (iii) inner PtNPs catalytic layer for propulsion (for details, see the Experimental Section). GO/Ni/PtNP MMs displayed the structural morphology based on a conical shape with dimensions of 5 μm width and 10 μm length. EDX mapping confirmed the elemental composition of the MMs homogeneously distributed (C as the sensing layer, Ni as the magnetic layer, and Pt as the catalytic layer), demonstrating the efficiency of the MM electrosynthesis.

**Figure 2:**
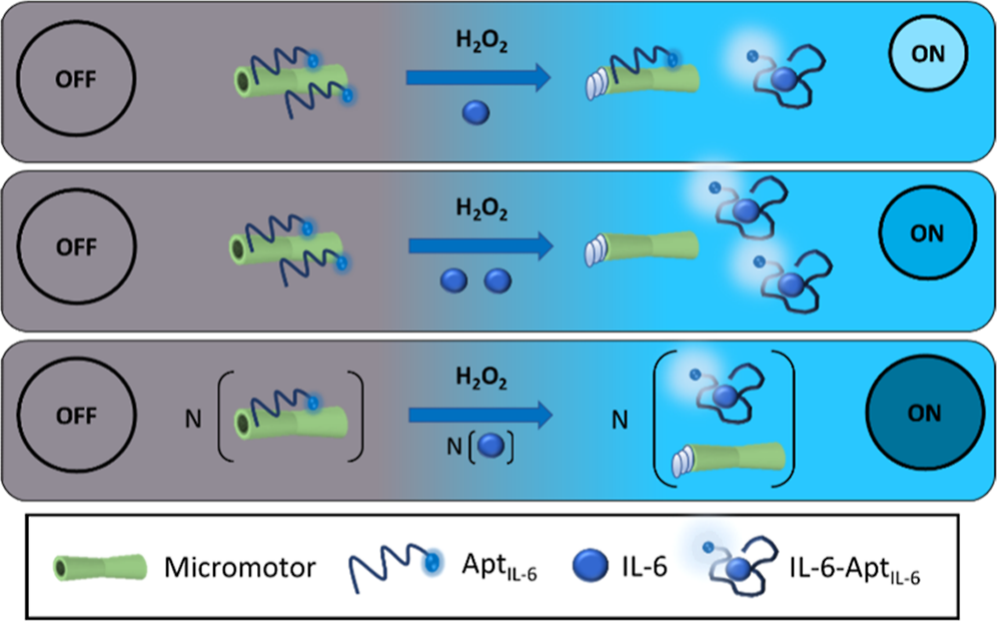
MM-based aptassay for the determination of IL-6. Optimization of the MM-based aptassay.

Figure 2 (Video S1) shows the analytical principle of this approach based on an OFF–ON fluorescent strategy, where highly specific IL-6 aptamers (Apt_IL-6_), labeled with a fluorophore, are quenched by their adsorption to the GO outer layer of MMs (MM_GO_–Apt_IL-6_). The presence of IL-6 in the sample implies the specific recognition between the aptamer and the protein (IL-6–Apt_IL-6_), producing a change in the structural conformation, which implies the separating from the MMs, and hence the recovering of the fluorescence signal, which is dependent on the concentration of protein in solution.

Before the on-the-move recognition of IL-6 molecules, an optimized quantity of MMs was incubated with an amount of the fluorophore-labeled aptamer to obtain the formation of π–π stacking interactions between GO and the nucleotide bases of the aptamer, which leads to the quenching of the fluorescence provided by the labeled aptamer (OFF state). Subsequently, a solution of IL-6 was added to the MMs with the fuel reagent (H_2_O_2_) to get the autonomous propulsion of MMs for the on-the-move recognition and capture of IL-6 molecules. Then, the specific affinity interaction with the aptamer (IL-6–Apt_IL-6_) produces a structural change in the 3D structure, providing the breaking of the MM_GO_–Apt_IL-6_ and recovering the fluorescence, which is concentration-dependent (ON state).

**Figure 3:**
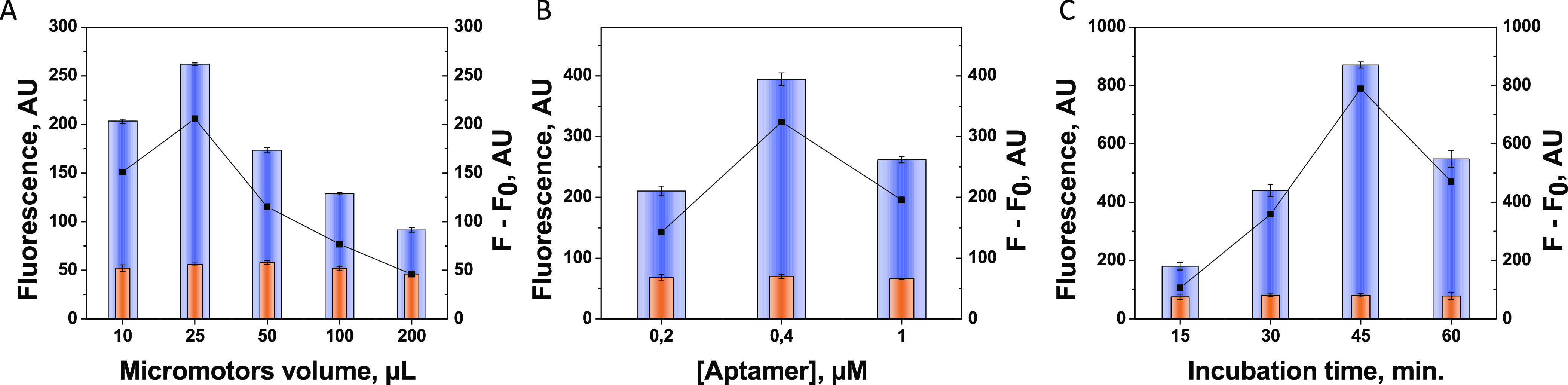
Optimization of the OFF state variables (formation of MM_GO_–Apt_IL-6_): volume of MM (A), aptamer concentration (B), and adsorption time (C). Controls without IL-6 (in orange).

Figure 3 shows the optimization of the variables involved in the formation of MM_GO_–Apt_IL-6_ (OFF state): number of MMs (A), aptamer concentration (B), and adsorption time (C).

MM number was first evaluated (see Figure 3A) to bind the adequate amount of aptamer that provides a suitable signal in the clinically relevant range of IL-6. To this end, different volumes of MMs (10 to 200 μL) with an excess of the specific aptamer (1 μM) and a fixed excess of IL-6 (2 μg mL^–1^) were explored. The optimum value of MMs was 25 μL (it corresponds to an MM number of 50,000 approx), where there is sufficient MM surface to adsorb enough aptamer molecules and to get the best performance. Larger amounts of MM give rise to aggregations that imply a lower active surface, reducing the possibility of π–π bond formation between the GO layer and the aptamer. In addition, higher amounts of MM produce an increase of O_2_ during the moving test due to the catalytic reaction after the addition of H_2_O_2_ in the Pt layer of MM produces O_2_, reducing the fluorescence. To immobilize MM_GO_–Apt_IL-6_, the aptamer concentration was then evaluated in a range of 0.2–1 μM to obtain the highest signal value, using the optimal MM number through stirring incubation. Figure 3B shows that the optimum value was 0.4 μM, while higher values displayed a lower signal probably due to a steric impediment produced by the excess of the aptamer on the MM surface.

The adsorption time of MM_GO_–Apt_IL-6_ was also studied, being 45 min the optimal value, while an excess of time probably causes the separation from the MM surface by excessive agitation (see Figure 3C).

**Figure 4:**
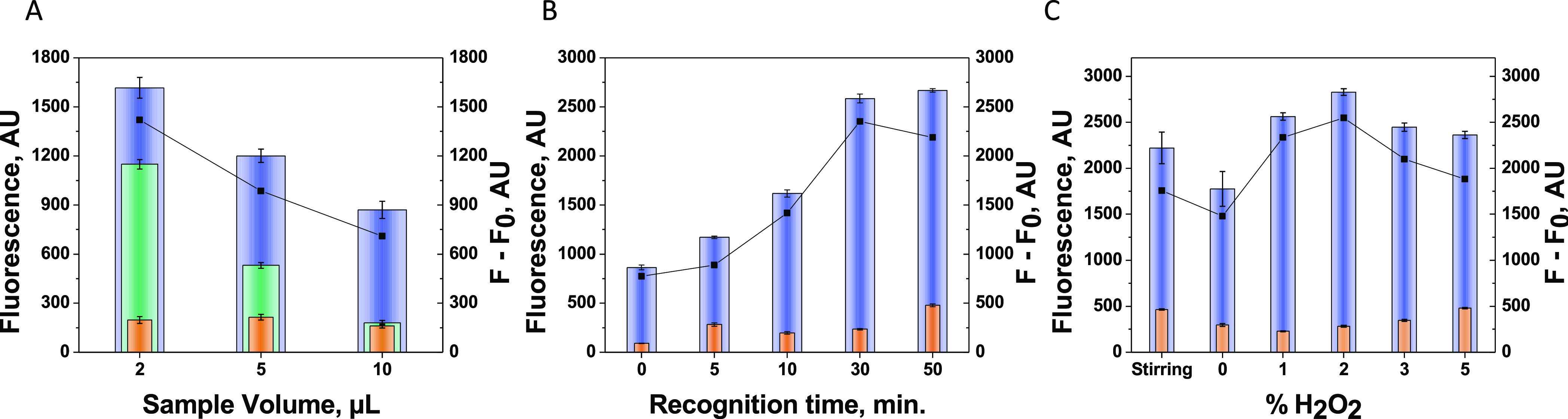
Optimization of the on-the-move aptassay (ON state): sample volume (A), recognition time (B), and propulsion conditions (C). Other conditions: 5% of BSA. Controls without IL-6 (in orange).

Figure 4 shows the optimization of the variables involving the aptassay by itself (ON state): sample volume (A), affinity reaction time (formation of the IL-6–Apt_IL-6_ complex and desorption of MM_GO_–Apt_IL-6_) (B), and fuel concentration (C). First, to avoid the physical adsorption of IL-6 directly to the MM surface, preventing its interaction with the immobilized aptamers, and hence decreasing sensitivity, the use of BSA during the on-the-move IL-6 aptassay was evaluated in the range of 1–10%. 5% of BSA was the selected value for sensitivity improvement.

Sample volume was carefully assayed as one of the key aptassay parameters. Interestingly, Figure 4A shows that just 2 μL of sample volume allowed one to obtain the highest sensitivity. Higher sample volumes dilute the concentration of the aptamer–IL-6 complex in solution, decreasing the fluorescence and hence avoiding reaching the low cut-off value (20 pg mL^–1^) for the diagnostic of the disease. The on-the-move affinity interaction time for IL-6 recognition was also evaluated. As it can be observed in Figure 4B, 30 min was the required time to form enough quantities of IL-6–Apt_IL-6_ due to the low volume of sample used, and, therefore, the low probability of interaction between the aptamer and the few IL-6 molecules. Longer times do not result in a significant improvement, and a shorter time is not enough time to complete the on-the-move recognition.

Propulsion is also a key factor in the development of the MM-based bioassays. Hydrogen peroxide levels as fuel was also assayed as a key factor in the propulsion behavior (Figure 4C). Hydrogen peroxide level was assayed between 0 and 5%, where 2% of H_2_O_2_ was considered optimum. At a higher level of H_2_O_2_, a poor behavior was noticed due to the increase of bubble formation, which hardly allowed the recognition event. Also, comparing the stirring movement or static conditions, the MM propulsion capabilities exhibited an improved performance at an optimized 2% of H_2_O_2_ with an enhanced sensitivity.

**Figure 5:**
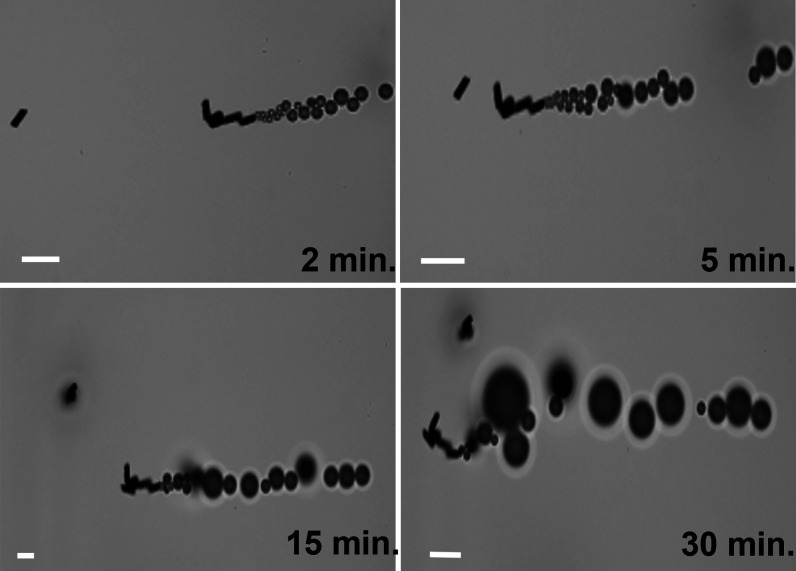
Time-lapsed microscopy images of MM navigation (taken from Video S2). Scale bar: 40 μm.

MMs were also able to move during the whole on-the-move assay time, showing their high propulsion capabilities even when the sample volume was extremely low (2 μL), resulting in the great efficiency of the assay, despite some visible aggregation due to its magnetism (Figure 5 and Video S2).

The optimized variables of the MM-based aptassay are summarized in Table 1, both the range studied and the selected value.

**Table 1:**
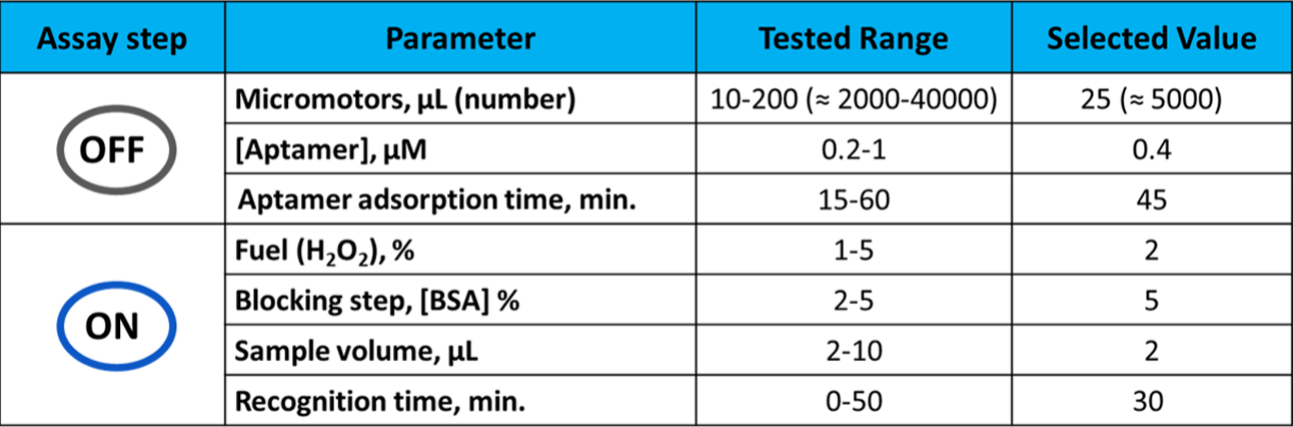
Optimized MM-Based Aptassay for IL-6

### Analytical Performance of the MM-Based Aptassay and Sample Analysis

**Figure 6:**
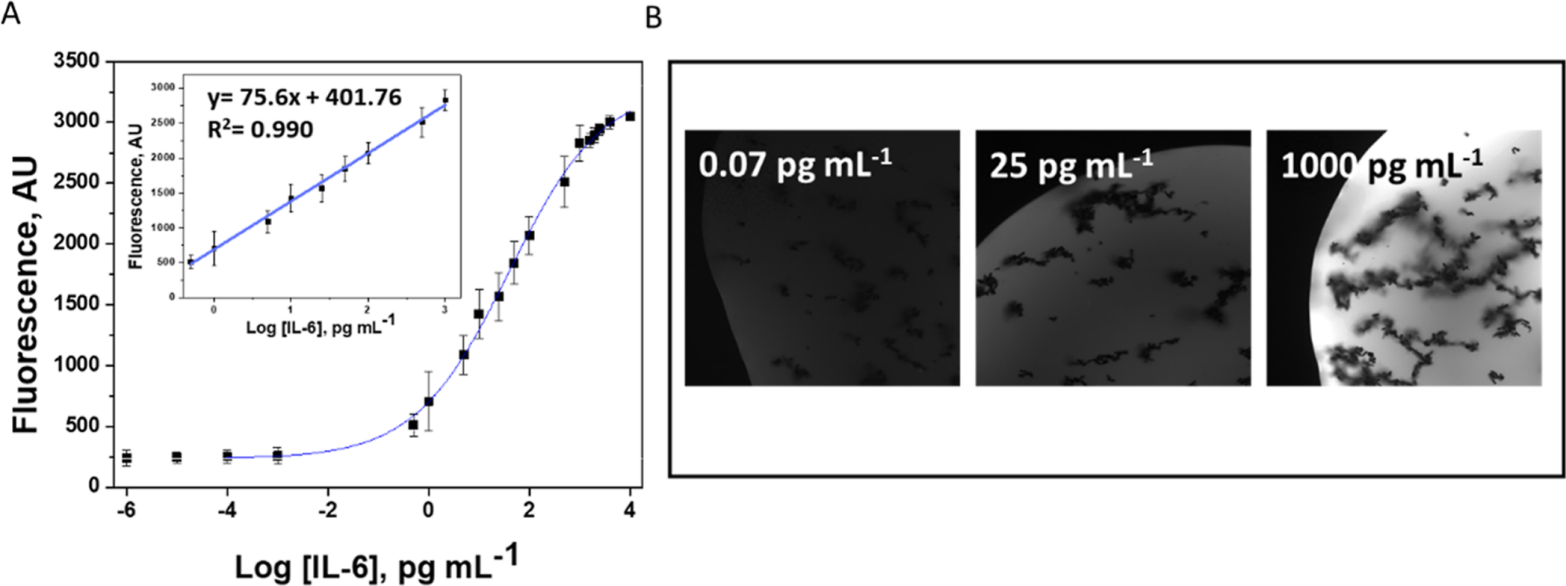
Sigmoidal curve and linear calibration (inset) plots (A) and fluorescence recovery images at selected concentrations of IL-6 (B). Other conditions were as in Figure 4.

Analytical performance of the MM-based aptassay was carefully evaluated. Figure 6A shows the calibration plots of IL-6–Apt_IL-6_. The calibration performance was excellent with a linear range of 0.07–1000 pg mL^–1^ (*r* = 0.995) covering the cut-off and clinical practice levels as well as exhibiting an impress sensitivity with LOD = 0.02 pg mL^–1^ and LOQ = 0.07 pg mL^–1^. The precision was also very good in both, at the minimum (0.07 pg mL^–1^) and maximum (1000 pg mL^–1^) IL-6 concentrations with values of RSD_0.07_ < 2% and RSD_1000_ < 3% (*n* = 10). Figure 6B shows the optical fluorescence images corresponding to the minimum (0.07 pg mL^–1^), EC_50_ (25 pg mL^–1^), and maximum (1000 pg mL^–1^) IL-6 concentrations, where the dependence of the fluorescence signal on the concentration is observed.

Finally, the MM-based aptassay was tested toward the analysis of clinical samples from very low birth weight previously diagnosed (*SepsisStaphylococcus aureus*, *Staphylococcus epidermis*, *Klebsiella pneumoniae*). Table 2 lists the IL-6 levels obtained using the MM-based aptassay and the reference values from the hospital. Interestingly, a high agreement between IL-6 levels obtained from our approach and the Hospital method (chemiluminescent immunoassay using cobas e411 analyzer) (30 μL of sample, 18 min, and LOD = 1.5 pg mL^–1^) was obtained (*E*_r_ < 3%). The MM-based aptassay is very competitive in comparison with the traditional one, in terms of a significant reduction of sample, 15 times less, and enhanced sensitivity, employing similar analysis times.

**Table 2:** Analysis of the Clinical Samples from Neonates with Very Low Birth Weight

patient number	patient clinical features	MM-based aptassay (pg mL^–1^)	hospital reference pg mL^–1^)	*E*, (%)
1	gestational age: 26 + 6 weeks, birthweight: 950 g, days of life: 55, Staphylococcus aureus	3.3 ± 0.3	3.3	0.4
2	gestational age: 24 + 5 weeks, birthweight: 830 g, days of life: 22, *Staphylococcus epidermis*	1060 ± 20	1076	1.5
3	gestational age: 26 + 6 weeks, birthweight: 970 g, days of life: 11, *Staphylococcus epidermis*	41.0 ± 1.6	40	1.6
4	gestational age: 23 + 3 weeks, birthweight: 616 g, days of life: 12, *Staphylococcus epidermis*	100.0 ± 0.8	100.5	0.9
5	gestational age: 26 + 0 weeks, birthweight: 990 g, days of life: 49, *Klebsiella pneumoniae*	33.0 ± 0.1	32.4	2.5
Healthy control	gestational age: 26 + 0 weeks, birthweight: 990 g, days of life: 22	1.50 ± 0.01	<1.5	0.5

Our approach constitutes the first MM reported in the literature for the determination of IL-6. Compared to other works found in the literature of aptassays developed for the determination of IL-6,^37,38,57−60^ it can be said that our approach allows to obtain one of the highest sensitivities found in the literature (LOD = 0.02 pg mL^–1^), allowing the determination of IL-6 in unique clinical samples, which are reported here for the first time, giving value to MM technology for diagnostic tests. These sensitivity values obtained are even more significant if the low volume of the sample used is considered, which is the smallest one (2 μL) reported.^37,38,59^ On the contrary, the recognition times were somewhat higher than those reported in other works,^38,57−60^ but this cannot be identified as a weakness since they are of the same order. Furthermore, it is important to indicate that this may be due to the low volume of sample tested. In addition, although the speed barely diminishes from the buffer (80 μm s^–1^) to serum samples (60 μm s^–1^), and some MM aggregation was observed due to the ultra-small volumes used, this fact does not affect the great capabilities of MM for the analysis of clinical samples, as it is clearly demonstrated in the results listed in Table 2. All this leads to the positioning of our MM-based aptassay as a competitive biosensing approach for the determination of IL-6 as a relevant marker of neonatal sepsis.

## Conclusions

The MM-based aptassay exhibited excellent biosensing capabilities for IL-6 determination in clinical samples from neonates with very low birth weight. The OFF–ON assay through an on-the-move recognition of IL-6 molecules was performed with a very low volume of sample (2 μL) with high sensitivity (LOD = 0.02 pg mL^–1^) and accuracy (*E*_r_ < 3%). Even more importantly, the linear range covered the clinical levels, and it allowed the direct determination without any dilution and simplifying the analysis.

There is no question that MM technology has become a potential analytical tool for low sample-based diagnostics in the fields where samples are hardly available. These results also identify the analytical potency of MMs for the development of multiplexed analysis. Other studies are currently under development in our lab in these directions. A new era has started to draw in the diagnostics landscape.

## Supporting Information Available

Principle of the current approach based on an OFF–ON fluorescent strategy (MP4)
Time lapse during MM navigation (MP4)

## Abbreviations

MMs: micromotors
IL-6: interleukin-6
*E*_r_: relative error
LOD: limit of detection
LOQ: limit of quantification
ICU: intensive care unit
CRP: C-reactive protein
PCT: procalcitonin
RSD: relative standard deviation
SEM: scanning electronic microscopy
EDX: energy-dispersive X-ray spectroscopy
